# Phenotype-driven management of diabetic macular edema: multimodal imaging biomarkers and individualized therapy

**DOI:** 10.3389/fmed.2026.1859928

**Published:** 2026-06-23

**Authors:** Zichao You, Li Jiang, Ying-rui Liu, Ming Ming Yang

**Affiliations:** 1 Department of Ophthalmology, Shenzhen People's Hospital, The Second Clinical Medical College, Jinan University, Shenzhen, China; 2 Department of Ophthalmology, Shenzhen People's Hospital (The First Affiliated Hospital, Southern University of Science and Technology), Shenzhen, China

**Keywords:** anti-VEGF therapy, diabetic macular edema, imaging biomarkers, intravitreal corticosteroids, phenotype-driven management

## Abstract

Diabetic macular edema (DME) remains a leading cause of vision impairment worldwide. Although intravitreal anti-vascular endothelial growth factor (anti-VEGF) agents are widely regarded as first-line treatment, a substantial proportion of patients demonstrate suboptimal responses, ranging from anatomical non-resolution to functional plateau, reflecting the marked pathophysiological heterogeneity of the disease. Multimodal retinal imaging, particularly optical coherence tomography (OCT), OCT angiography (OCTA), and ultra-widefield fluorescein angiography (UWF-FA)— has enabled detailed characterization of retinal structural and microvascular alterations and facilitated the identification of clinically relevant imaging biomarkers. This review synthesizes established and emerging imaging biomarkers and groups them into two major domains. First, markers of disease activity, represented by intraretinal and subretinal fluid and hyperreflective foci. Second, predictors of visual prognosis, including disorganization of the retinal inner layers, photoreceptor damage, and retinal perfusion deficits. Crucially, based on characteristic biomarker profiles, we propose stratifying DME into five principal clinical phenotypes: the leakage-dominant, inflammatory, tractional, focal-treatment, and poor-prognosis phenotypes. Integrating these phenotypes into proposed, hypothesis-generating decision-support framework—encompassing baseline assessment, phenotype-based stratification, and dynamic optimization, aims to align therapeutic strategies to be more precisely aligned with underlying pathogenic mechanisms. Future developments, including automated biomarker quantification and artificial intelligence-assisted image analysis, may further enhance precision in DME phenotyping and support a definitive shift towards truly individualized disease management.

## Introduction

Diabetic macular edema (DME) is a leading cause of visual impairment among working-age individuals with diabetes mellitus (1). Although the advent of intravitreal anti–vascular endothelial growth factor (anti-VEGF) therapy has substantially transformed the therapeutic landscape of DME and established anti-VEGF agents as the standard first-line treatment, large randomized clinical trials, including Protocol T, as well as real-world studies have consistently demonstrated that approximately 30–50% of patients exhibit suboptimal anatomical or functional responses or require frequent injections to maintain disease control (2, 3). This includes anatomical non-response (persistent fluid) and functional plateau (poor vision despite resolved edema). Such an empirical treatment paradigm not only increases healthcare burden but may also delay optimal visual recovery in those who could benefit from alternative therapeutic strategies. The therapeutic complexity of DME largely reflects its marked pathophysiological heterogeneity, encompassing disruption of the blood–retinal barrier, chronic inflammatory activation, neurodegeneration, and vitreomacular traction (4–6). Conventional management strategies have predominantly relied on quantitative changes in central subfield thickness (CST), often overlooking the distinct biological mechanisms underlying fluid accumulation. With the widespread adoption of multimodal imaging techniques, including optical coherence tomography (OCT), OCT angiography (OCTA), and ultra-widefield fluorescein angiography (UWF-FA), clinicians are now able to characterize microstructural alterations and retinal perfusion status with increasing precision (7). In this context, imaging biomarkers that reflect specific pathological processes may provide a more refined basis for therapeutic stratification. The aim of the present review is therefore to summarize current evidence on key imaging biomarkers relevant to individualized management of DME and to discuss how these markers may be integrated into an imaging phenotype–based approach to support treatment selection and therapeutic switching.

## Literature search strategy and evidence considerations

This clinically oriented narrative review was informed by targeted literature searches of PubMed, Embase, the Cochrane Library and Web of Science for studies published up to March 2026. Search terms combined diabetic macular edema–related terminology with retinal imaging modalities and biomarker-specific keywords, including optical coherence tomography, optical coherence tomography angiography, ultra-widefield fluorescein angiography, intraretinal fluid, subretinal fluid, hyperreflective foci, disorganization of the retinal inner layers, deep capillary plexus, foveal avascular zone, epiretinal membrane, and vitreomacular traction. Reference lists of relevant original studies and recent reviews were also manually screened to identify additional publications. Studies were excluded if they were case reports, conference abstracts, editorials or non-human investigations lacking direct clinical relevance to DME. Studies were considered eligible if they evaluated structural or microvascular imaging biomarkers in relation to DME pathophysiology, disease activity, visual prognosis, treatment response, or longitudinal progression. Priority was given to randomized clinical trials (RCTs), multicenter prospective studies, longitudinal observational cohorts, *post hoc* analyses of landmark trials, and systematic reviews where available. Exploratory single-center observational studies were also included when addressing emerging biomarkers for which higher-level evidence remains limited.

Given the heterogeneity of imaging acquisition protocols, biomarker definitions, and study endpoints across the literature, we established a structured grading system to interpret the evidence according to study design, methodological robustness, and consistency of reported clinical associations. Evidence was categorized into three tiers: high-level (e.g., large-scale multicenter RCTs and well-conducted systematic reviews or meta-analyses with direct clinical impact), moderate-level (e.g., longitudinal cohort studies, *post hoc* analyses of landmark trials, and replicated single-center prospective studies), and low to exploratory-level (e.g., derived primarily from exploratory retrospective studies, small single-center OCTA cohorts, or preliminary artificial intelligence analyses). To provide a structured overview of the current evidence landscape, the principal imaging biomarkers discussed in this review and their corresponding levels of supporting evidence are summarized in Table 1. These evidence considerations provide context for the biomarker-based phenotypic framework discussed in the following sections.

**Table 1:** Current clinical evidence supporting major multimodal imaging biomarkers in diabetic macular edema.

Biomarker category	Evidence level (*)	Study design & rationale	Representative references
CST & fluid distribution (IRF/SRF)	High	Validated by large-scale, multicenter RCTs and long-term extensions. Primary endpoints for clinical decision-making.	(2, 3, 13)
HRF	Moderate	Supported by meta-analyses and longitudinal cohorts; however, quantification standards remain variable.	(22, 24, 26)
VMIA (VMT, ERM, VMA)	Moderate	Prospective and retrospective observational studies evaluating structural traction, treatment response, and surgical outcomes in DME.	(36, 38)
Structural integrity (DRIL, EZ/ELM)	Moderate	Robust predictors of functional recovery with high spatial correlation to visual outcomes in clinical cohorts.	(26, 40, 41, 43)
UWF-FA peripheral biomarkers (NPI, PPL)	Moderate	Prospective studies linking peripheral ischemia and lesions to DME progression and treatment burden.	(65–67)
Chronic remodeling (ORTs, ICHRM)	Low to exploratory	Based on small-scale case series and retrospective observational data on treatment-resistant or long-standing DME cases.	(55–57, 59)
OCTA perfusion metrics (DCP-VD, FAZ)	Low to exploratory	Primarily derived from single-center cross-sectional studies. Limited by significant inter-device algorithmic heterogeneity and lack of standardized measurement pipelines.	(61, 62, 64)

*Evidence level definitions: High: Validated by multicenter RCTs or high-quality meta-analyses with direct clinical impact. Moderate: Consistent findings from multiple longitudinal, prospective, or retrospective observational cohorts. Low to exploratory: Findings from single-center cohorts, cross-sectional studies, or preliminary exploratory parameters; requires further large-scale validation before broad clinical implementation. CST = central subfield thickness; IRF = intraretinal fluid; SRF = subretinal fluid; RCT = randomized controlled trial; HRF = hyperreflective foci; VMIA = vitreoretinal interface abnormalities; VMT = vitreomacular traction; ERM = epiretinal membrane; VMA = vitreomacular adhesion; DRIL = disorganization of the retinal inner layers; EZ = ellipsoid zone; ELM = external limiting membrane; UWF-FA = ultra-widefield fluorescein angiography; NPI = non-perfusion index; PPL = predominantly peripheral lesions; ORTs = outer retinal tubulations; ICHRM = intracystic hyperreflective material; OCTA = optical coherence tomography angiography; DCP-VD = deep capillary plexus vessel density; FAZ = foveal avascular zone.

## Imaging biomarkers informing clinical decision-making in DME

### Biomarkers reflecting disease activity and underlying pathobiology

#### Patterns of retinal fluid distribution

Retinal fluid accumulation represents the hallmark pathological feature of diabetic macular edema (DME) and reflects disruption of the blood–retinal barrier (BRB) with subsequent vascular extravasation (8). Based on anatomical location on OCT, fluid can be categorised as intraretinal fluid (IRF) or subretinal fluid (SRF). The morphology, laminar distribution, volume, and dynamic changes of these fluid compartments are critical parameters for assessing disease activity and guiding therapeutic decision-making. IRF typically manifests as cystoid macular edema (CME) or sponge-like diffuse retinal thickening (DRT) (9). It is predominantly associated with breakdown of the inner BRB, and its volume correlates directly with the degree of vascular leakage (10). Studies by Chang et al. and Somagani et al. demonstrated that IRF serves as a robust biomarker of DME activity. Baseline IRF is significantly associated with increased central subfield thickness (CST) and impaired visual function, whereas resolution of IRF following treatment parallels CST reduction. However, excessive cystic enlargement (diameter > 100 μm) has been linked to poorer visual prognosis (11, 12).

SRF, in contrast, arises from disruption of the outer BRB and choroidal inflammatory involvement, presenting as serous retinal detachment (SRD) on OCT (13). Multiple studies have shown that the presence of SRF is frequently associated with elevated aqueous inflammatory cytokines, including interleukin-6 (IL-6), suggesting an inflammation-driven disease phenotype. Baseline SRF has been identified as an independent and strong predictor of CST reduction and significant visual improvement following corticosteroid therapy (13, 14). The coexistence of IRF and SRF indicates heightened disease activity and appears to confer increased responsiveness to corticosteroid treatment. Furthermore, dynamic fluid shifts—such as reduction of IRF with concomitant increase in SRF after anti-VEGF therapy—may suggest insufficient inflammatory control and warrant consideration of combination or alternative therapeutic strategies (14).

#### Müller cell dysfunction and neurovascular unit impairment

Conventional assessment of DME commonly uses intraretinal and subretinal fluid as indicators of disease activity. However, these fluid compartments largely represent downstream consequences of severe BRB disruption (5). Recent work has shifted focus upstream to the neurovascular unit (NVU), and in particular to early cellular stress and dysfunction of Müller cells. Lai et al. described cytoplasmic swelling of Müller cells as an early change in DME, manifesting on OCT as sponge-like diffuse retinal thickening (DRT), consistent with cellular stress induced by hypoxia and inflammation. With disease progression, failure of Müller cell ion and water channels [notably Kir4.1 and aquaporin-4 (AQP4)] and subsequent liquefactive necrosis lead to the formation of cystoid macular edema (CME), yielding an imaging continuum of Müller-cell dysfunction (15). Spaide and Szeto have further demonstrated that the spatial distribution of CME closely overlaps areas of ischemia and non-perfusion within the deep capillary plexus (DCP), and that OCTA can visualize DCP flow abnormalities with high fidelity. These observations support a model in which inadequate energy supply from the DCP precipitates failure of Müller cell fluid transport (pump failure), representing a proximate pathological mechanism for retinal fluid accumulation (16, 17). Wang et al. (18) have emphasized that Müller cell-derived VEGF is an important driver of vascular leakage and local inflammation, exacerbating BRB breakdown and DCP ischemia; on fluorescein angiography this process commonly manifests as widespread, diffuse leakage. In summary, imaging signatures of Müller cell dysfunction link ischemia, inflammation, and structural disruption in a mechanistic chain. This ischemia-driven Müller cell pump failure may partly explain why eyes with predominant DCP dropout show limited response to pure anti-VEGF therapy (19). When interpreted alongside OCTA assessment of DCP perfusion, these cellular-level and microcirculatory markers may refine assessment of DME activity beyond fluid distribution alone.

#### Hyperreflective foci and inflammatory biomarkers

Retinal hyperreflective foci (HRF) are discrete, punctate lesions on OCT with a diameter < 30 μm and reflectivity approaching that of the retinal nerve fiber layer (RNFL). They are thought predominantly to represent activated microglia and constitute a quantitative marker of inflammatory burden and disease activity in DME (20). Multiple studies have reported significant positive correlations between baseline HRF burden and aqueous humor inflammatory mediators, including interleukin-6 (IL-6) and monocyte chemoattractant protein-1 (MCP-1) as well as systemic inflammatory indices such as the neutrophil-to-lymphocyte ratio (NLR) and the systemic immune-inflammation index (SII), consistent with a high-activity inflammatory phenotype (21, 22).

Anatomical and pathological specificity of HRF distribution has been described. Shu et al. (23) showed that inner-layer HRF, located between the internal limiting membrane (ILM) and the inner nuclear layer (INL), do not correlate significantly with CST or the presence of SRF, conversely outer-layer HRF, situated from the outer plexiform layer (OPL) to the retinal pigment epithelium (RPE), correlate closely with SRF formation and area—findings that are compatible with migration of activated microglia towards the outer retina and consequent disruption of the outer BRB. Fragiotta et al. (24) further reported that, in progressive disease, HRF migrate outward accompanied by disruption of the external limiting membrane (ELM) and the ellipsoid zone (EZ); importantly, the decline in HRF number after treatment often precedes visible edema resolution, supporting HRF as a sensitive biomarker of disease progression and therapeutic responsiveness.

From a therapeutic perspective, studies by Vujosevic et al. (25, 26) demonstrated that a high baseline HRF load characterizes an inflammatory DME phenotype that tends to respond better anatomically and functionally to corticosteroid treatment than to anti-VEGF therapy; accordingly, a reduction in HRF count is a useful indicator of anti-inflammatory treatment effect and may guide personalized therapy selection. In contrast, a meta-analysis by Nanji et al. (27) found that a high baseline HRF burden is independently associated with limited visual benefit after treatment, identifying HRF as a negative prognostic marker for visual outcome. Advances in automated quantification have facilitated more objective assessment: Wang et al. (28) developed a deep-learning method for HRF quantification with a reported accuracy of 82.3%, and further systematic evaluation of HRF volume, density and distance to the foveal center is likely to enhance the role of HRF in inflammatory phenotyping and therapeutic decision-making.

**Figure 1:**
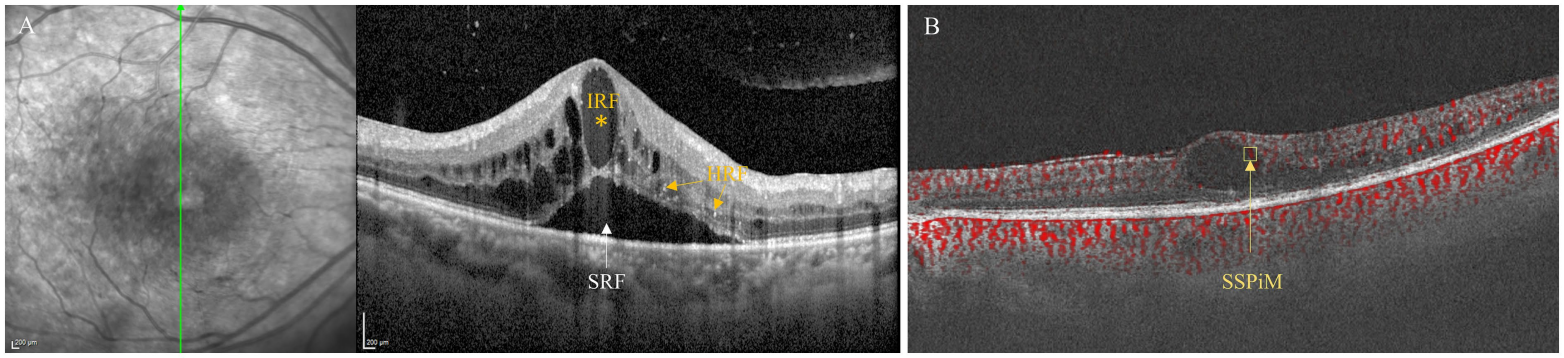
Multimodal imaging of the inflammatory DME phenotype. **(A)** Optical coherence tomography (OCT) B-scan demonstrates key markers of active inflammation: severe intraretinal fluid (IRF) as cystoid macular edema (CME, asterisks), subretinal fluid (SRF, white arrowhead) indicating outer blood-retinal barrier breakdown, and numerous hyperreflective foci (HRF, yellow arrows). **(B)** OCT angiography (OCTA) B-scan showing suspended scattering particles in motion (SSPiM, yellow arrows) within cystoid spaces, a biomarker of stagnant fluid and high inflammatory load. Clinical images were obtained from de-identified patients at Shenzhen People’s Hospital under Institutional Ethics Committee approval (no. LL-KY-2024016-01).

In addition to HRF, suspended scattering particles in motion (SSPiM) are recognized on OCTA as non-vascular, high-reflectivity signals arising from the motion of lipid and protein particles within intraretinal cystic spaces; they represent severe BRB disruption and may act as a marker of treatment-resistant disease. Genç et al. (29) and Zhou et al. (30) have observed that SSPiM localize to regions of microvascular abnormality and often predict a poor anatomical response to anti-VEGF monotherapy; in some cases, SSPiM subsequently evolve into hard exudates on follow-up. Clinically, SSPiM can artefactually elevate OCTA-derived vessel density (VD) measurements. Therefore, VD overestimation due to SSPiM should be corrected, and clinicians should avoid misinterpreting a post-treatment VD decrease (caused by resolution of SSPiM) as an increase in ischemia. SSPiM should also be regarded as a refractory biomarker that may signal limited efficacy of anti-VEGF monotherapy and prompt consideration of alternative or combination anti-inflammatory strategies (Figure 1).

#### Vascular leakage and capillary perfusion abnormalities

Vascular leakage represents an important dimension in the assessment of DME activity. Markan et al. reported that leakage patterns on FFA reflect varying degrees of BRB disruption. Focal leakage is most commonly attributable to microaneurysms (MAs) and may serve as an indication for focal laser photocoagulation. In contrast, diffuse leakage frequently accompanies widespread capillary dysfunction and disorganization of the retinal inner layers (DRIL), suggesting a higher inflammatory burden and concomitant ischemia, and may signal progressive disease (31).

Parravano et al. (32) demonstrated that MAs localized to the DCP on OCTA constitute an independent risk factor for the progression of retinal fluid accumulation, implying greater leakage potential in this subset of lesions. Maltsev et al. further showed that structural en face OCT enables accurate identification of angiographically leaking MAs, which are often characterized by distinct morphological alterations. These findings support the use of non-invasive imaging approaches for the assessment of leakage activity (33). Hein et al. (34) reported that angiographic characteristics are significantly associated with both anatomical and functional responses following anti-VEGF therapy, underscoring the relevance of vascular pathological status in evaluating DME treatability and disease activity.

#### Vitreoretinal interface abnormalities and traction

Vitreomacular interface abnormalities (VMIA), encompassing vitreomacular traction (VMT), epiretinal membrane (ERM) and vitreomacular adhesion (VMA), represent key mechanical drivers in the pathogenesis of DME. From a mechanistic perspective, Hui et al. (35) reported that tractional forces generated by VMT may disrupt the internal limiting membrane (ILM), promoting the release of VEGF and inflammatory mediators and exacerbating microvascular dysfunction. In a prospective study, Shrivastava et al. further demonstrated that VMIA was significantly negatively correlated with baseline best-corrected visual acuity (BCVA) and occurred more frequently in patients with proliferative diabetic retinopathy (PDR), particularly in those with elevated glycated hemoglobin (HbA1c) levels and a diabetes duration exceeding 10 years. These findings suggest that VMIA may serve as a marker of chronicity in DME (36).

With regard to therapeutic response and prognostic stratification, several studies have identified VMIA as a strong predictor of reduced responsiveness to pharmacological therapy. Chatziralli et al. (37) and Costanzo et al. (14), in studies evaluating anti-VEGF therapy and dexamethasone intravitreal implants, respectively, observed significantly lower anatomical and functional response rates in eyes with baseline ERM or VMT. Murakami et al. (38) emphasized that OCT enables accurate identification of ERM and VMT, and highlighted that DME eyes with pronounced VMIA may exhibit suboptimal outcomes with pharmacotherapy alone, warranting alternative management strategies beyond conventional medical pathways. Building on this concept, Guo et al. proposed in a randomized controlled trial design that minimally invasive pars plana vitrectomy (PPV) combined with ILM peeling may be considered as an initial treatment option in treatment-naïve DME. This strategy aims to relieve mechanical traction at an early stage and facilitate the clearance of inflammatory mediators, thereby potentially mitigating pharmacological resistance and reducing subsequent retreatment burden (39). Prince et al. further noted that postoperative visual gain in DME depends on residual retinal functional reserve. Extensive preoperative DRIL or disruption of the EZ and ELM may constrain visual improvement, underscoring the need for multidimensional biomarker integration in surgical decision-making. Although ILM peeling itself may not directly enhance visual acuity, it appears to reduce ERM recurrence and may therefore be more appropriate in eyes with ILM thickening or ERM involvement of the foveal center (40).

#### Imaging determinants of visual prognosis and functional recovery

Accurate assessment of visual prognosis in DME remains a central clinical challenge. A frequent observation in routine practice is the phenomenon of structural–functional dissociation, whereby a reduction in CST does not translate into proportional visual acuity improvement. This discrepancy highlights the limitations of conventional evaluation strategies relying primarily on BCVA and CST measurements, which may not adequately reflect the upper limit of functional recovery. Visual prognosis in DME is determined not only by reversible fluid resorption but also by the extent of irreversible neuronal damage, disruption of the outer retinal architecture, and the severity of retinal ischemia. Consequently, the identification of robust imaging biomarkers capable of predicting functional outcomes is of considerable clinical importance. Multimodal imaging, including OCT, OCTA, and FFA, provides complementary structural and vascular information. The integration of these imaging modalities offers multidimensional support for prognostic stratification, the optimization of therapeutic expectations, and the individualization of treatment strategies.

#### Inner retinal structural integrity and disorganization

Neurodegeneration in DME predominantly involves the inner retina and is characterized by disruption of the neuronal signal transmission. On OCT, DRIL and ganglion cell complex (GCC) atrophy represent key structural biomarkers. DRIL is defined as the loss or indistinctness of the normal laminar boundaries between the inner plexiform layer (IPL), inner nuclear layer (INL), and ganglion cell layer (GCL) within the central 1 mm of the fovea (41). In a longitudinal study, Sun et al. (41) demonstrated that DRIL outperformed CST in predicting visual outcomes; a greater baseline DRIL extent within the central 1 mm was associated with a poorer visual outcome, with each 300 μm increase in DRIL corresponding to a one-line reduction in visual acuity. Das et al. (42) further quantified this association, reporting that every 100 μm increase in DRIL extent was associated with a decrease of 4.6 ETDRS letters. A meta-analysis by Nanji et al. (27) confirmed that eyes with baseline DRIL exhibited a mean loss of 7.3 ETDRS letters at 12 months, with DRIL involving the foveal center exerting a more pronounced adverse effect on visual acuity. OCTA-based analyses by Moein et al. (43) and Cennamo et al. (44) revealed a high spatial concordance between DRIL regions and reductions in superficial capillary plexus vessel density (SCP-VD) and deep capillary plexus vessel density (DCP-VD), as well as an enlargement of the foveal avascular zone (FAZ), implicating macular ischemia as a principal driver of DRIL formation. Tripathi et al. (45) reported that an intravitreal dexamethasone implant may partially reverse DRIL, thereby providing a rationale for therapeutic adjustment in refractory cases.

GCC atrophy is characterized by thinning of the retinal nerve fiber layer (RNFL), GCL, and IPL. In a comparative study of macular edema secondary to DME and central retinal vein occlusion (CRVO), Mathurkar et al. (46) demonstrated significantly reduced GCC thickness in the DME cohort relative to the CRVO group. Conversely, Condelipes et al. (47) reported in patients with persistent DME that GCC thickness within the central 1 mm was moderately negatively correlated with BCVA, suggesting that pathological thickening of the GCC during the chronic edematous phase may also portend poorer visual outcomes. Compared with DRIL, which reflects structural disorganization, GCC atrophy represents a more advanced quantitative indicator of inner retinal neuronal damage. Longitudinal assessment of GCC thickness may therefore serve as a biomarker for evaluating neuroprotective efficacy and long-term visual prognosis in DME.

#### Outer retinal biomarkers of photoreceptor integrity

Outer retinal biomarkers constitute a central dimension in evaluating visual prognosis and guiding individualized treatment strategies in DME. The EZ and ELM, which serve as critical anatomical barriers of the photoreceptor complex, have structural continuity that directly determines the potential for visual recovery. Maheshwary et al. (48) quantitatively demonstrated a significant negative correlation between the percentage of EZ disruption and BCVA; for every 1% increase in the extent of EZ loss, mean visual acuity decreased by 0.3 ETDRS letters. Studies by Santos et al. (49) and Kessler et al. (50) further identified baseline EZ integrity as an independent predictor of long-term visual gain, with the restoration of the EZ during treatment closely paralleling improvements in visual acuity. From a mechanistic perspective, Saxena et al. (51) reported that elevated VEGF levels induce sequential disruption of the ELM followed by the EZ, whereas under anti-VEGF therapy, recovery of the ELM precedes that of the EZ. These findings underscore the role of ELM integrity as a prerequisite structural scaffold for photoreceptor regeneration. Tsai et al. (52) observed that macular ischemia significantly exacerbates EZ disruption and that the duration of EZ damage is more predictive of 12-month visual outcomes than baseline status alone, suggesting that macular ischemia may potentiate this irreversible degenerative process.

Photoreceptor outer segment (PROS) length, defined as the distance between the EZ and the RPE, represents a quantitative marker of photoreceptor integrity. Abd Elhamid (53) demonstrated that PROS length was significantly reduced in eyes with DME compared with healthy controls, and that its correlation with visual acuity was stronger than that of CST. Ozkaya et al. (54) further confirmed a stepwise reduction in PROS thickness with increasing severity of diabetic retinopathy (DR), with PROS demonstrating a significant positive correlation with visual acuity. In addition, Sardana et al. (55) and Santos et al. (49) reported that preserved baseline EZ and ELM integrity and greater PROS length were associated with more favorable anatomical resolution and visual improvement following anti-VEGF therapy. Collectively, quantitative OCT-based assessment of EZ and ELM integrity, together with PROS length, enables the precise delineation of retinal functional reserve and may assist in estimating the potential benefit of anti-VEGF therapy in DME.

#### Biomarkers of chronic retinal remodeling

With progression to the chronic stage of DME, the retina undergoes structural remodeling accompanied by functional decompensation. Intracystic hyperreflective material (ICHRM) and outer retinal tubulations (ORTs) have emerged as imaging biomarkers indicative of chronicity. ICHRM refers to discrete hyperreflective material located within cystoid spaces or along the cyst wall in the foveal region on OCT, and is thought to be associated with cyst fibrosis or glial proliferation (56). Venkatesh et al. (57) demonstrated that eyes with baseline ICHRM had significantly poorer final BCVA and required 4.7-fold and 6.2-fold more anti-VEGF and corticosteroid injections, respectively, identifying ICHRM as a strong predictor of treatment resistance. Upadhyaya et al. (56) further reported that ILM peeling combined with intraoperative OCT-guided cyst puncture improved BCVA from 20/71.4 to 20/40 in such eyes, without recurrence, thereby providing a surgical rationale for pharmacologically refractory cases.

**Figure 2:**
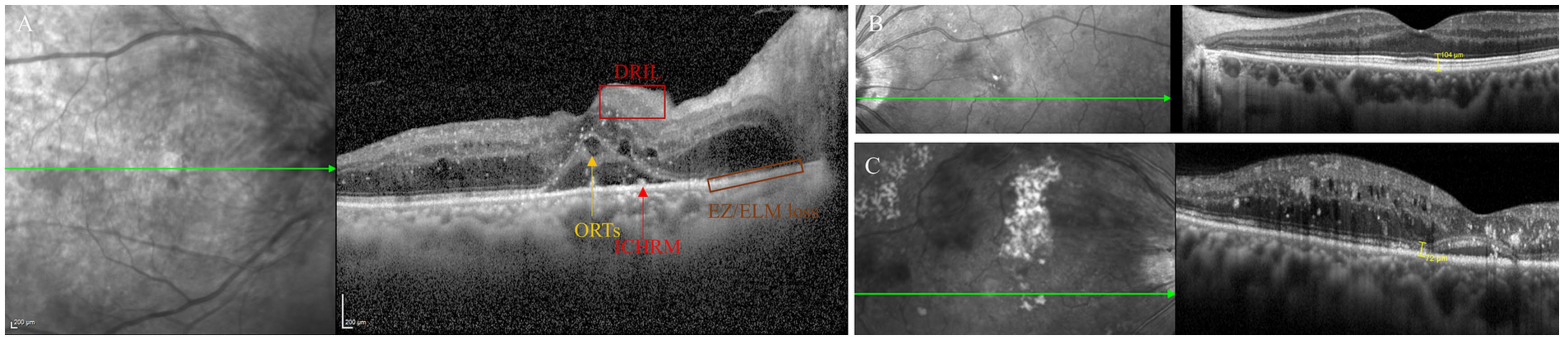
Structural OCT biomarkers predicting poor visual prognosis and chronic remodelling. **(A)** OCT illustrating multiple biomarkers of structural damage and chronicity, including extensive disorganization of the retinal inner layers (DRIL, red box), disruption of the external limiting membrane (ELM) and ellipsoid zone (EZ) (brown box), which reflect loss of photoreceptor integrity. Chronic markers include intracystic hyperreflective material (ICHRM, red arrow) and an outer retinal tubulation (ORT, yellow arrow), the latter representing advanced rearrangement of degenerating photoreceptors. **(B,C)** Intra-patient comparison reveals a preserved photoreceptor outer segment (PROS) length (104 μm) in the non-DME eye **(B)**, compared with a markedly reduced PROS length (72 μm) in the fellow DME eye **(C)**, reflecting quantitative photoreceptor loss. Clinical images were obtained from de-identified patients at Shenzhen People’s Hospital under Institutional Ethics Committee approval (no. LL-KY-2024016-01).

ORTs represent a late-stage manifestation of neuroglial remodelling secondary to chronic photoreceptor injury. Huang et al. (58) reported a prevalence of approximately 24.8% in eyes undergoing anti-VEGF therapy, with ORTs typically located within the outer nuclear layer (ONL) or outer plexiform layer (OPL), persistent ORTs were significantly associated with poorer final visual acuity and the disruption of photoreceptor integrity. Al-Halafi (59) and Espina et al. (60) emphasized that ORTs do not respond to anti-VEGF therapy and should be distinguished from active CME in order to avoid overtreatment. Collectively, the identification of ICHRM and ORTs on OCT facilitates the recognition of chronic retinal remodeling in DME and may assist in distinguishing pharmacological resistance from irreversible structural degeneration, thereby informing appropriate therapeutic decision-making (Figure 2).

#### Macular ischemia and perfusion abnormalities

Ischemia-related biomarkers are central to elucidating microvascular dysfunction in DME, and the non-invasive, layer-resolved capability of OCTA provides a robust platform for their quantitative assessment. Mirshahi et al. (61) demonstrated that, compared with DR eyes without DME, eyes with DME exhibited more pronounced perfusion deficits in both the SCP and the DCP. Lee et al. (19) further reported that reductions in DCP-VD, rather than SCP-VD, were more closely associated with a suboptimal response to anti-VEGF therapy. Basiony et al. (62) subsequently quantified this relationship, showing that treatment response rates were significantly improved when central foveal DCP-VD was ≥ 27.5% and parafoveal DCP-VD was ≥ 44.05%, suggesting that structural disruption of the DCP may impair the efficiency of intraretinal fluid resolution.

Enlargement of the foveal avascular zone (FAZ) serves as a surrogate marker of macular ischemia. Studies by Hein et al. (34) and Santamaría et al. (63) reported that eyes with greater baseline FAZ enlargement or reduced vessel density demonstrated limited visual improvement despite effective CST reduction following anti-VEGF therapy. These findings indicate that established microvascular ischemia may be largely irreversible and highlight the importance of baseline perfusion assessment in delineating the potential for visual recovery.

Neovascularization (NV), representing an advanced manifestation of severe ischemia, can also be quantitatively evaluated using OCTA. Ishibazawa et al. (64) proposed that OCTA enables precise measurement of optic disc NV flow area and allows dynamic monitoring of regression and recurrence following treatment, thereby providing an objective basis for individualizing retreatment intervals.

#### Peripheral retinal alterations on ultra-widefield imaging

**Figure 3:**
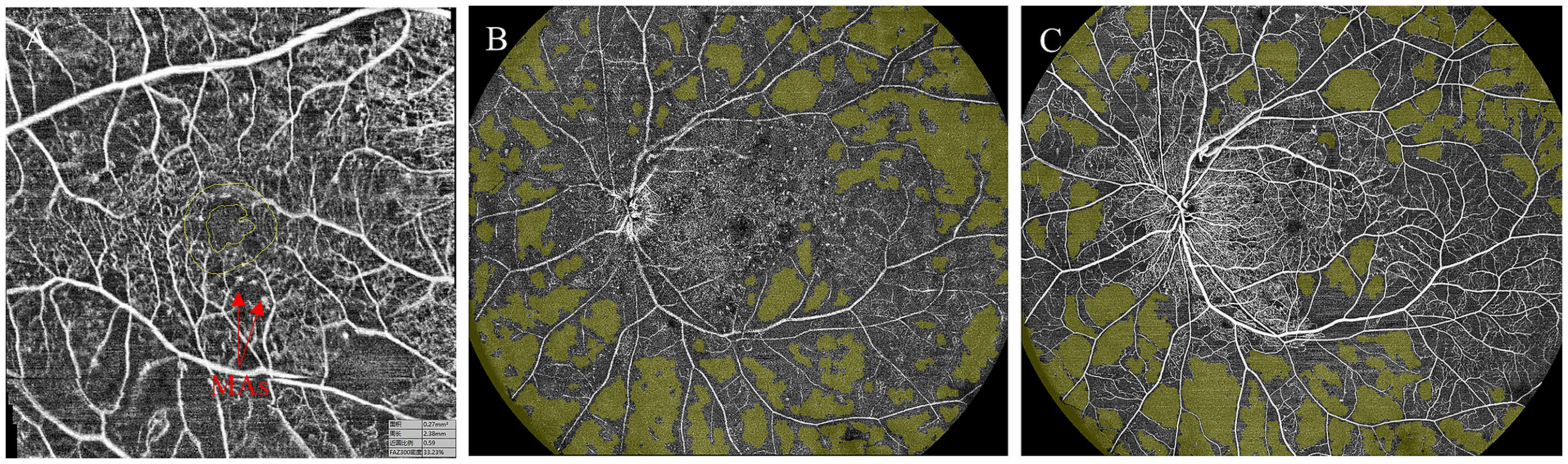
OCTA biomarkers of macular ischemia in DME. **(A)** 6 × 6 mm En face OCTA showing an enlarged foveal avascular zone (FAZ, yellow outline) and microaneurysms (MAs, red arrows). **(B,C)** The deep capillary plexus (DCP) **(B)** exhibits severe capillary dropout, whereas the superficial capillary plexus (SCP) **(C)** remains relatively preserved, demonstrating that ischaemic damage in DME is typically more pronounced in the DCP. Clinical images were obtained from de-identified patients at Shenzhen People’s Hospital under Institutional Ethics Committee approval (no. LL-KY-2024016-01).

Ultra-widefield (UWF) imaging has overcome the field-of-view limitations of conventional modalities, thereby refining current understanding of DME pathophysiology and informing clinical management. Using UWF fluorescein angiography (UWF-FA), Wessel et al. (65) demonstrated that peripheral retinal ischemia promotes BRB disruption through the upregulation of VEGF, conferring a 3.75-fold increased risk of DME development. In a longitudinal cohort, Silva et al. further quantified this association, reporting that for every 0.1-unit increase in the retinal non-perfusion index (NPI), the risk of DME progression increased by 11%. Moreover, eyes with predominantly peripheral lesions (PPL) exhibited a 1.89-fold greater risk of disease worsening compared with PPL-negative eyes. Notably, both parameters were independent of baseline Diabetic Retinopathy Severity Scale (DRSS) score and significantly enhanced prognostic stratification (66). Patel et al. (67) further confirmed that eyes with refractory DME displayed a significantly elevated ischemia index (ISI), with the extent of retinal ischemia positively correlated with the degree of resistance to anti-VEGF therapy. From a structural perspective, Chiku et al. (68) employed swept-source OCT (SS-OCT) to achieve the simultaneous quantification of macular and peripheral retinal edema, demonstrating that, in DME eyes, both macular volume and peripheral retinal volume were significantly increased, and that these parameters escalated in parallel with worsening non-proliferative diabetic retinopathy (NPDR) severity. Regarding therapeutic prediction and guidance, Jiang et al. developed a random forest model showing that the panretinal leakage index and ISI independently predicted future anti-VEGF injection frequency, with an area under the receiver operating characteristic curve (AUC) of 0.91 for predicting immediate treatment requirement (69), thereby providing an objective basis for treatment burden estimation. Morel et al. (70) reported that although anti-VEGF therapy may improve DRSS grading, it does not reverse established retinal non-perfusion. Rabiolo et al. (71) further emphasized that the identification of peripheral ischemia is of particular importance in guiding targeted retinal photocoagulation (TRP) to reduce VEGF load (Figure 3).

## Translating imaging phenotypes into individualized therapy

### Conceptual integration of multimodal imaging biomarkers

Given the marked heterogeneity of DME pathophysiology, reliance on a single imaging biomarker is insufficient to comprehensively characterize disease behavior and may limit the implementation of precision-based therapeutic strategies. Accordingly, clinical evaluation should move beyond isolated parameter interpretation towards an integrated phenotyping framework based on multimodal imaging. One representative integrative approach is the “TCED-HFV” scoring system, originally described by Panozzo et al. (72) within the ESASO spectral-domain OCT grading framework. This composite tool incorporates seven structural parameters, each weighted by severity, to provide an objective basis for identifying eyes with reduced anti-VEGF responsiveness: (i) T (Thickening): Central subfield thickness (scored 0–2); (ii) C (Cysts): Cystoid macular edema (0–3); (iii) E (EZ/ELM status): Photoreceptor integrity (0–2); (iv) D (DRIL): Inner retinal disorganization (0–1); (v) H (Hyperreflective foci): (0–1); (vi) F (Subretinal fluid): (0–1); (vii) V (Vitreoretinal relationship):(0–4). A composite score of ≥12 points (maximum 14) is highly specific for poor anti-VEGF response. The original publication presents the detailed grading criteria in a consolidated tabular format, which readers may consult directly (72). To enhance clinical applicability, and building upon the pathological relevance and therapeutic implications of the core biomarkers discussed above, we further propose an integrated framework structured around principal pathogenic dimensions. On this basis, DME may be categorized into five core clinical phenotypes (Table 2): the leakage-dominant phenotype, the inflammatory phenotype, the tractional phenotype, the focal-treatment phenotype, and the poor-prognosis phenotype. It should be emphasized that the quantitative thresholds presented in Table 2 represent a synthesis of currently available evidence from specific single-center studies using particular imaging devices, scan protocols, and segmentation algorithms. They are intended to facilitate phenotype conceptualization and clinical reasoning rather than to serve as universal, device-agnostic decision cut-offs. Absolute values vary significantly across OCT/OCTA platforms (e.g., 3 × 3 mm vs. 6 × 6 mm scans), and inter-device algorithmic heterogeneity limits direct transferability. Prospective, multicenter validation studies are warranted to further establish their clinical utility; clinicians should validate these thresholds locally and interpret them within the full clinical context before application.

**Table 2:** Multimodal imaging–based phenotypic classification of DME and guidance for individualised management.

Clinical phenotype	Key imaging biomarkers (*)	Primary pathophysiological mechanisms	Therapeutic strategies and clinical guidance	Measurement context & key caveats
Leakage-dominant phenotype	① OCT: IRF (CME/DRT), no significant SRF or HRF, intact EZ/ELM; ② OCTA: DCP vessel density ≥ 27.5%, increased Mas; ③ FFA: Focal or diffuse leakage, no obvious ischaemia.	VEGF-mediated inner BRB disruption, impairment of vascular endothelial tight junctions, fluid extravasation, and Müller cell fluid transport dysfunction.	Anti-VEGF agents as first-line therapy. T&E regimen following the initial loading phase; follow-up OCT every 8–12 weeks, adjusting intervals based on changes in CST and IRF.	DCP-VD ≥ 27.5%: Derived from RTVue-XR Avanti (Optovue), 6 × 6 mm scan, automated full-retinal-thickness segmentation (62).
Inflammatory phenotype	① OCT: SRF present, > 20 outer retinal HRF; ② OCTA: Reduced DCP vessel density; ③ FFA: Diffuse intense hyperfluorescent leakage.	Inflammatory cytokines (e.g., IL-6, MCP-1) driving outer BRB and RPE disruption; predominantly an inflammatory burden.	Corticosteroid implants preferred; follow-up OCT every 4–6 weeks to monitor the resolution of SRF and HRF.	HRF > 20: Total count of outer-layer HRF within central 1 mm foveal zone on spectral-domain OCT B-scans. Based on manual/semi-automated enumeration (20, 26, 28)
Tractional phenotype	① OCT: VMT (traction ≥ 1,500 μm), tractional ERM involving the fovea, non-resolving IRF post-treatment.	Mechanical stress activates Müller cells to release VEGF and IL-6, impeding fluid absorption and drug diffusion.	PPV with ERM peeling ± ILM peeling; post-operative anti-VEGF therapy as needed, with follow-up OCT at 1, 3, and 6 months.	VMT ≥ 1,500 μm: threshold derived from morphological classification systems; clinical significance also depends on associated retinal distortion and EZ integrity.
Focal-treatment phenotype	① OCT: No diffuse IRF, intact EZ/ELM; ② OCTA: No obvious macular ischaemia; ③ FFA: Focal MAs (> 500 μm from the fovea centre), leakage area < 10 mm^2^.	Localised vascular barrier breakdown at individual MAs; circumscribed leakage, with no significant inflammation or ischaemia.	Precision focal laser photocoagulation; follow-up FFA at 3 months, with supplementary anti-VEGF injections if leakage persists.	MAs > 500 μm from fovea: distance measured on OCTA en face or FFA relative to foveal center. Leakage area <10 mm^2^: Quantified on early-phase FFA (30–60 s). Both parameters require standardized field-of-view and magnification calibration.
Poor-prognosis phenotype	① OCT: DRIL > 1,000 μm, disrupted EZ/ELM, SSPiM, GCC atrophy, ICHRM/ORTs; ② OCTA: DCP vessel density < 25%, FAZ area > 0.4 mm^2^; ③ UWF-FA: NPI > 0.18, PPL positive.	NVU impairment, ischaemia-driven neurodegeneration, and irreversible photoreceptor damage.	Focus on maintaining visual function; consider adjunctive neuroprotective therapy; regular OCT/OCTA monitoring for RPE atrophy progression; combine TRP in cases with peripheral non-perfusion.	DRIL >1,000 μm: Central 1 mm on SD-OCT (41, 42). DCP-VD < 25% + FAZ > 0.4 mm^2^: SD-OCTA, device-specific (62, 63). NPI > 0.18: Optos UWF-FA (66), prognostic only.

(*) All quantitative thresholds presented in this table are study-derived examples reported in the cited literature and are not intended as universal, device-agnostic clinical decision cut-offs. IRF = intraretinal fluid; CME = cystoid macular oedema; DRT = diffuse retinal thickening; SSPiM = suspended scattering particles in motion; HRF = hyperreflective foci; SRF = subretinal fluid; VMT = vitreomacular traction; ERM = epiretinal membrane; PPV = pars plana vitrectomy; MAs = microaneurysms; FAZ = foveal avascular zone; DCP = deep capillary plexus; DRIL = disorganisation of the retinal inner layers; EZ = ellipsoid zone; ELM = external limiting membrane; ICHRM = intracystic hyperreflective material; ORTs = outer retinal tubulations; NPI = non-perfusion index; PPL = predominantly peripheral lesions; T&E = treat-and-extend; BRB = blood–retinal barrier; RPE = retinal pigment epithelium; NVU = neurovascular unit; TRP = targeted retinal photocoagulation; ILM = internal limiting membrane.

### Mixed and transitional phenotypes: treatment-priority logic and re-phenotyping

While the five-phenotype framework provides a structured conceptual foundation, clinical practice frequently presents overlapping biomarker profiles that defy singular classification. Eyes with vitreomacular traction may simultaneously exhibit inflammatory biomarkers such as HRF or SRF, whereas inflammatory phenotype may coexist with poor-prognosis features DRIL, ellipsoid zone disruption, or deep capillary plexus ischaemia. Accordingly, phenotypic classification should be regarded as a dynamic and integrative process rather than a rigid categorical framework.

In mixed presentations, therapeutic interpretation may follow a hierarchical framework based on pathogenic reversibility and immediate clinical actionability. When clinically significant vitreomacular interface abnormalities are present, including vitreomacular traction or epiretinal membrane associated with structural distortion, tractional factors may warrant primary consideration, as persistent mechanical stress may perpetuate edema and limit pharmacological responsiveness. In such cases, PPV may be considered in selected eyes. Postoperative reassessment at 4–8 weeks is recommended to determine whether residual inflammatory or exudative activity warrants adjunctive intravitreal therapy. Notably, limited evidence suggests that vitrectomy accelerates the clearance of intravitreal anti-VEGF agents, which may necessitate more frequent dosing to maintain therapeutic efficacy (73). Conversely, the clinical outcomes and pharmacokinetic profiles of corticosteroid therapy appear to show no significant difference between vitrectomized and non-vitrectomized eyes (74, 75).

In the absence of dominant tractional pathology, therapeutic decisions are more appropriately guided by the predominant active component. Eyes characterised by diffuse IRF accompanied by inflammatory biomarkers may initially respond to anti-vascular endothelial growth factor therapy. However, persistent exudative activity following an initial treatment phase may justify reassessment of the dominant pathological component and consideration of alternative treatment strategies. Emerging prospective studies have further suggested that selected treatment-naïve eyes with pronounced inflammatory burden, including abundant HRF, serous retinal detachment, or hard exudates, may derive greater anatomical benefit and reduced treatment burden from early combination approaches incorporating anti-VEGF agents and intravitreal corticosteroids, although current evidence remains limited (76–78).

Poor-prognosis biomarkers, including DRIL, photoreceptor disruption, and macular ischaemia, should not be interpreted as direct treatment-triggering factors but rather as modifiers of expected functional recovery. Their presence may instead inform patient counselling, realistic visual expectations, and individualised retreatment goals. Longitudinal phenotypic reassessment therefore remains particularly important when biomarker profiles evolve during treatment.

Taken together, mixed and transitional phenotypes appear to be common in DME. The hierarchical principles outlined above should therefore be regarded as a clinically informed interpretive framework rather than a prescriptive treatment algorithm. Given that several biomarker-treatment associations remain derived predominantly from retrospective or exploratory studies, prospective multicentre validation will be required before formal integration into routine clinical practice.

### Phenotype-guided treatment stratification and optimization

**Figure 4:**
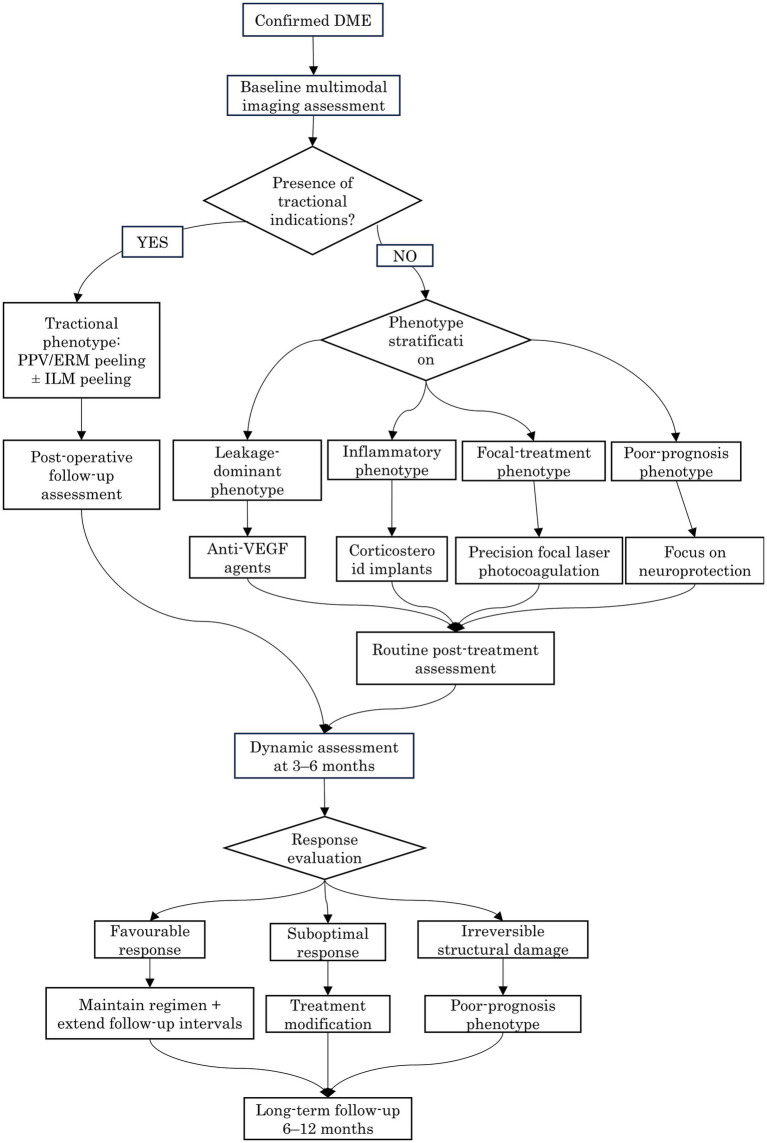
Flowchart for individualized DME decision-making based on multimodal imaging biomarkers. (1) Baseline assessment: Multimodal imaging identifies tractional indications, prompting pars plana vitrectomy (PPV) with ERM/ILM peeling. (2) Phenotype stratification (non-tractional): The leakage-dominant phenotype receives first-line anti-VEGF therapy, including conventional agents, faricimab, and the ranibizumab port delivery system (PDS). The inflammatory phenotype is preferentially treated with intravitreal corticosteroids. The focal-treatment phenotype undergoes focal laser photocoagulation. The poor-prognosis phenotype is managed with neuroprotective goals and adjunctive targeted retinal photocoagulation for peripheral non-perfusion. (3) Dynamic optimization: At 3–6 months, favourable response permits follow-up extension; suboptimal response warrants treatment modification only after structural confirmation on OCT/OCTA excludes non-DME causes; irreversible structural damage prompts reclassification to the poor-prognosis phenotype with overtreatment avoidance. Sensitivity caveats: baseline CST < 300 μm is evaluated by IRF/HRF dynamics; inflammatory or tractional cases may require earlier reassessment at 4–8 weeks.

Building upon the integrative assessment of multimodal imaging biomarkers, this review proposes a structured decision-support framework for guiding individualized DME management, consisting of three sequential stages, pending prospective validation: baseline assessment, phenotype-based stratification, and dynamic optimization (Figure 4). This framework is designed to provide a clinically actionable decision-support tool. (1) Baseline assessment: A comprehensive baseline evaluation integrates OCT, OCTA, FFA, and relevant systemic parameters. Priority is given to identifying surgical indications for the tractional phenotype. In cases meeting the criteria for mechanical traction, PPV is performed, followed by adjunctive anti-VEGF therapy as required. (2) Phenotype-based stratification: In the absence of tractional indications, patients are stratified according to core imaging biomarkers. The leakage-dominant phenotype is managed with intravitreal anti-VEGF agents as first-line therapy, encompassing conventional options (bevacizumab, ranibizumab, aflibercept), the bispecific angiopoietin-2/VEGF-A inhibitor faricimab, and sustained-release formulations such as the ranibizumab port delivery system (PDS). Loading injections are typically administered monthly for 3–6 doses, followed by a treat-and-extend (T&E) regimen; transition to corticosteroid or combination therapy should be considered if substantial IRF persists alongside elevated HRF or SRF after loading. The inflammatory phenotype is preferentially treated with intravitreal corticosteroid therapy, though in phakic eyes or those with borderline glaucoma, anti-VEGF monotherapy may be preferred and IOP monitoring is mandatory if corticosteroids are used. The focal-treatment phenotype undergoes targeted focal laser photocoagulation. For the poor-prognosis phenotype, management is central on neuroprotection and functional preservation with structured 2–3 months follow-up; TRP is an active intervention for peripheral non-perfusion, not a fallback. (3) Dynamic optimization: Treatment response is reassessed dynamically after 3–6 months. A reduction in CST of ≥20% without DRIL progression is defined as a favorable response, supporting the extension of follow-up intervals. Conversely, a CST reduction of < 10% or a decline of ≥ 5 ETDRS letters indicates a suboptimal response (3, 76), warranting treatment modification. In the presence of irreversible structural damage, patients are reclassified under the poor-prognosis phenotype, with management goals shifted towards the maintenance of existing visual function and the strict avoidance of overtreatment. Clinicians should note several sensitivity considerations: (i) eyes with baseline CST < 300 μm may not achieve a 20% reduction even with effective therapy, and should be evaluated primarily by IRF resolution and HRF dynamics; (ii) ETDRS letter decline may reflect cataract, media opacity, or coexistent retinal pathology rather than DME worsening; (iii) the 3–6 month reassessment window is appropriate for leakage-dominant phenotypes, but inflammatory or tractional cases with ongoing damage may require earlier reassessment at 4–8 weeks to prevent permanent structural loss, and (iv) any treatment modification prompted by a ≥ 5-letter ETDRS decline must be preceded by concurrent structural confirmation on OCT or OCTA, ensuring that the functional loss is consistent with active disease activity rather than non-DME pathology.

## Limitations, future directions and conclusions

### Limitations

Several limitations should be acknowledged when interpreting the proposed phenotype framework. First, quantitative biomarker thresholds including DCP vessel density, FAZ area, and HRF counts are derived predominantly from single-center studies using specific OCT/OCTA devices and scan protocols, and their generalizability across platforms remains uncertain. Second, the cited literature is heavily weighted toward East Asian and European cohorts, potentially limiting applicability to other ethnic groups. Third, the proposed dynamic optimization criteria represent pragmatic clinical syntheses rather than prospectively validated thresholds, requiring multicenter confirmation. Fourth, the five-phenotype model simplifies the biological continuum of DME; although a hierarchical logic for mixed presentations is proposed, formal prospective validation of treatment-priority sequencing is still lacking. Finally, as a narrative review, this work did not employ a systematic review methodology with dual independent screening; however, structured searches, explicit inclusion criteria, and three-tier evidence grading were applied to enhance transparency.

### Future directions

Therapeutic options for macular ischemia and neurodegeneration remain insufficient. Promising neuroprotective strategies include brimonidine implants, somatostatin analogues, minocycline, and PKC-*β* inhibitors (77–79). Anti-ischemia approaches target HIF-1α and Notch1 pathways (80), while Tie2/Angiopoietin activators may promote vascular repair beyond anti-VEGF blockade (81). Complement modulation (e.g., C3 inhibitors) and cellular/gene therapies (CD34 + stem cells, AAV-mediated anti-VEGF delivery) are under active investigation (82, 83). Systemically, GLP-1 receptor agonists and SGLT2 inhibitors show emerging retinal neurovascular protective effects (84). These modalities, though not yet standard care, underscore the trajectory toward neurovascular unit protection in phenotype-guided management.

Beyond therapeutic innovation, future efforts must prioritize imaging protocol standardization, multimodal predictive model development, and prospective multicenter validation of the phenotype framework. Automated biomarker quantification and AI-assisted analysis will be essential to reduce observer variability and enable truly individualized care.

### Conclusion

The management paradigm of DME is transitioning from empirical intervention toward phenotype-driven precision medicine. Through integrative multimodal imaging analysis, key biomarkers enable the construction of five principal clinical phenotypes: the leakage-dominant, inflammatory, tractional, focal-treatment, and poor-prognosis phenotypes. Within a proposed decision-support framework encompassing baseline assessment, phenotype stratification, and dynamic optimization, therapeutic strategies may be more precisely aligned with underlying pathogenic mechanisms, with the potential to improve visual outcomes whilst reducing unnecessary treatment burden. Overcoming the current limitations in microvascular repair and therapeutic targeting of neurodegeneration may ultimately facilitate the translation of phenotype-guided precision management from a conceptual framework to broad clinical implementation.

## References

[1] IDF. Diabetes Atlas. (2025). Available online at: https://diabetesatlas.org/resources/idf-diabetes-atlas-2025/ (Accessed December 5, 2025).

[2] Glassman AR Wells JA Josic K Maguire MG Antoszyk AN Baker C . Five-year outcomes after initial Aflibercept, bevacizumab, or Ranibizumab treatment for diabetic macular edema (protocol T extension study). Ophthalmology. (2020) 127:1201–10. doi: 10.1016/j.ophtha.2020.03.021, 32402554 PMC7483366

[3] Bressler NM Beaulieu WT Maguire GM Glassman AR Blinder KJ Bressler SB . Early response to anti-vascular endothelial growth factor and two-year outcomes among eyes with diabetic macular edema in protocol T. Am J Ophthalmol. (2018) 195:93–100. doi: 10.1016/j.ajo.2018.07.030, 30077569 PMC6648655

[4] Tsai T Kuehn S Tsiampalis N Vu MK Kakkassery V Stute G . Anti-inflammatory cytokine and angiogenic factors levels in vitreous samples of diabetic retinopathy patients. PLoS One. (2018) 13:e0194603. doi: 10.1371/journal.pone.0194603, 29584759 PMC5870958

[5] Antonetti DA Silva PS Stitt AW. Current understanding of the molecular and cellular pathology of diabetic retinopathy. Nat Rev Endocrinol. (2021) 17:195–206. doi: 10.1038/s41574-020-00451-4, 33469209 PMC9053333

[6] Romero-Aroca P Baget-Bernaldiz M Pareja-Rios A Lopez-Galvez M Navarro-Gil R Verges R. Diabetic macular edema pathophysiology: Vasogenic versus inflammatory. J Diabetes Res. (2016) 2016:1–17. doi: 10.1155/2016/2156273, 27761468 PMC5059543

[7] Parravano M Cennamo G Di Antonio L Grassi MO Lupidi M Rispoli M . Multimodal imaging in diabetic retinopathy and macular edema: an update about biomarkers. Surv Ophthalmol. (2024) 69:893–904. doi: 10.1016/j.survophthal.2024.06.006, 38942124

[8] Daruich A Matet A Moulin A Kowalczuk L Nicolas M Sellam A . Mechanisms of macular edema: beyond the surface. Prog Retin Eye Res. (2018) 63:20–68. doi: 10.1016/j.preteyeres.2017.10.006, 29126927

[9] Otani T Kishi S Maruyama Y. Patterns of diabetic macular edema with optical coherence tomography. Am J Ophthalmol. (1999) 127:688–93. doi: 10.1016/S0002-9394(99)00033-1, 10372879

[10] Zhang J Zhang J Zhang C Zhang J Gu L Luo D . Diabetic macular edema: current understanding, molecular mechanisms and therapeutic implications. Cells. (2022) 11:3362. doi: 10.3390/cells11213362, 36359761 PMC9655436

[11] Somagani R Malik M Somagani R Rakesh KK. A study of visual outcomes and spectral domain optical coherence tomography (SD-OCT) biomarker changes in patients treated with Ranibizumab for diabetic macular edema in a tertiary hospital. Cureus. (2025) 17:e82520. doi: 10.7759/cureus.82520, 40385737 PMC12085908

[12] Chang YC Huang YT Hsu AY Meng PP Lin CJ Lai CT . Optical coherence tomography biomarkers in predicting treatment outcomes of diabetic macular edema after Ranibizumab injections. Medicina (Kaunas). (2023) 59:629. doi: 10.3390/medicina59030629, 36984630 PMC10053215

[13] Zur D Iglicki M Busch C Invernizzi A Mariussi M Loewenstein A . OCT biomarkers as functional outcome predictors in diabetic macular edema treated with dexamethasone implant. Ophthalmology. (2018) 125:267–75. doi: 10.1016/j.ophtha.2017.08.031, 28935399

[14] Costanzo E Giannini D De Geronimo D Fragiotta S Varano M Parravano M. Prognostic imaging biomarkers in diabetic macular edema eyes treated with Intravitreal dexamethasone implant. J Clin Med. (2023) 12:1303. doi: 10.3390/jcm12041303, 36835839 PMC9968175

[15] Lai D Wu Y Shao C Qiu Q. The role of Müller cells in diabetic macular edema. Invest Ophthalmol Vis Sci. (2023) 64:8. doi: 10.1167/iovs.64.10.8, 37418272 PMC10337800

[16] Szeto SK Lai TY Vujosevic S Sun JK Sadda SR Tan G . Optical coherence tomography in the management of diabetic macular oedema. Prog Retin Eye Res. (2024) 98:101220. doi: 10.1016/j.preteyeres.2023.101220, 37944588

[17] Spaide RF. Retinal vascular cystoid macular edema: review and new theory. Retina. (2016) 36:1823–42. doi: 10.1097/IAE.0000000000001158, 27328171

[18] Wang J Xu X Elliott MH Zhu M Le YZ. Müller cell-derived VEGF is essential for diabetes-induced retinal inflammation and vascular leakage. Diabetes. (2010) 59:2297–305. doi: 10.2337/db09-1420, 20530741 PMC2927953

[19] Lee J Moon BG Cho AR Yoon YH. Optical coherence tomography angiography of DME and its association with anti-VEGF treatment response. Ophthalmology. (2016) 123:2368–75. doi: 10.1016/j.ophtha.2016.07.010, 27613201

[20] Vujosevic S Bini S Torresin T Berton M Midena G Parrozzani R . Hyperreflective retinal spots in normal and diabetic eyes: B-scan and En face spectral domain optical coherence tomography evaluation. Retina. (2017) 37:1092–103. doi: 10.1097/IAE.0000000000001304, 27668929

[21] Zhou J Song S Zhang Y Jin K Ye J. OCT-based biomarkers are associated with systemic inflammation in patients with treatment-naïve diabetic macular edema. Ophthalmol Ther. (2022) 11:2153–67. doi: 10.1007/s40123-022-00576-x, 36166152 PMC9587150

[22] Ergin E Dascalu AM Stana D Tribus LC Arsene AL Nedea MI . Predictive role of complete blood count-derived inflammation indices and optical coherence tomography biomarkers for early response to Intravitreal anti-VEGF in diabetic macular edema. Biomedicine. (2025) 13:1308. doi: 10.3390/biomedicines13061308, 40564027 PMC12189659

[23] Shu Y Zhang C Bi Y Zhang J. Hyperreflective foci and subretinal fluid predicts microglia activation involved in the breakdown of outer blood-retinal barrier in treatment-naïve patients with diabetic macular edema. Asia Pac J Ophthalmol (Phila). (2025) 14:100168. doi: 10.1016/j.apjo.2025.100168, 40043956

[24] Fragiotta S Abdolrahimzadeh S Dolz-Marco R Sakurada Y Gal-Or O Scuderi G. Significance of Hyperreflective foci as an optical coherence tomography biomarker in retinal diseases: characterization and clinical implications. J Ophthalmol. (2021) 2021:1–10. doi: 10.1155/2021/6096017, 34956669 PMC8709761

[25] Vujosevic S Toma C Villani E Muraca A Torti E Florimbi G . Diabetic macular edema with neuroretinal detachment: OCT and OCT-angiography biomarkers of treatment response to anti-VEGF and steroids. Acta Diabetol. (2020) 57:287–96. doi: 10.1007/s00592-019-01424-4, 31541333

[26] Vujosevic S Torresin T Bini S Convento E Pilotto E Parrozzani R . Imaging retinal inflammatory biomarkers after intravitreal steroid and anti-VEGF treatment in diabetic macular oedema. Acta Ophthalmol. (2017) 95:464–71. doi: 10.1111/aos.13294, 27775223

[27] Nanji K Grad J Hatamnejad A El-Sayes A Mihalache A Gemae M . Baseline OCT biomarkers associated with visual acuity in diabetic macular edema: a systematic review and Meta-analysis. Ophthalmology. (2025) 133:75–90. doi: 10.1016/j.ophtha.2025.07.038, 40803536

[28] Wang X Zhang Y Ma Y Kwapong WR Ying J Lu J . Automated evaluation of retinal hyperreflective foci changes in diabetic macular edema patients before and after intravitreal injection. Front Med (Lausanne). (2023) 10:1280714. doi: 10.3389/fmed.2023.1280714, 37869163 PMC10587607

[29] Genç G Yanık Ö Demirel S Batioglu F Özmert E. The longitudinal follow-up of a newly proposed OCTA imaging finding (SSPiM) and the importance of it as a new biomarker for treatment response in diabetic macular edema. Graefes Arch Clin Exp Ophthalmol. (2024) 262:2491–502. doi: 10.1007/s00417-024-06457-2, 38530451 PMC11271326

[30] Zhou L Sun H Li C Luo T Meng T Wen X . Topographic associations of hyperreflective materials in diabetic retinopathy: a multimodal correlation with microvascular pathology, structural remodeling and systemic metabolic dysregulation. Front Med (Lausanne). (2025) 12:1619819. doi: 10.3389/fmed.2025.1619819, 40740946 PMC12307468

[31] Markan A Agarwal A Arora A Bazgain K Rana V Gupta V. Novel imaging biomarkers in diabetic retinopathy and diabetic macular edema. Ther Adv Ophthalmol. (2020) 12:2515841420950513. doi: 10.1177/2515841420950513, 32954207 PMC7475787

[32] Parravano M De Geronimo D Scarinci F Virgili G Querques L Varano M . Progression of diabetic microaneurysms according to the internal reflectivity on structural optical coherence tomography and visibility on optical coherence tomography angiography. Am J Ophthalmol. (2019) 198:8–16. doi: 10.1016/j.ajo.2018.09.031, 30308201

[33] Maltsev DS Kulikov AN Burnasheva MA Kazak AA Chhablani J. Structural en face optical coherence tomography imaging for identification of leaky microaneurysms in diabetic macular edema. Int Ophthalmol. (2020) 40:787–94. doi: 10.1007/s10792-019-01239-w, 31797175

[34] Hein M Vukmirovic A Constable IJ Raja V Athwal A Freund KB . Angiographic biomarkers are significant predictors of treatment response to intravitreal aflibercept in diabetic macular edema. Sci Rep. (2023) 13:8128. doi: 10.1038/s41598-023-35286-2, 37208427 PMC10199070

[35] Hui VWK Szeto SKH Tang F Yang D Chen H Lai TYY . Optical coherence tomography classification Systems for Diabetic Macular Edema and Their Associations with Visual Outcome and treatment responses—an updated review. Asia Pac J Ophthalmol (Phila). (2022) 11:247–57. doi: 10.1097/APO.0000000000000468, 34923521

[36] Shrivastava N Som V Kumar K. Study of imaging biomarkers as a prognostic factor and guide in the Management of Diabetic Macular Oedema. Cureus. (2024) 16:e73765. doi: 10.7759/cureus.73765, 39677190 PMC11646637

[37] Chatziralli I Dimitriou E Lambadiari V Kazantzis D Kapsis P Theodossiadis G . The impact of laboratory findings and optical coherence tomography biomarkers on response to Intravitreal anti-VEGF treatment in patients with diabetic macular edema. Semin Ophthalmol. (2022) 37:668–75. doi: 10.1080/08820538.2022.2069470, 35468026

[38] Murakami T Ishihara K Terada N Nishikawa K Kawai K Tsujikawa A. Pathological neurovascular unit mapping onto multimodal imaging in diabetic macular edema. Medicina (Kaunas). (2023) 59:896. doi: 10.3390/medicina59050896, 37241128 PMC10221113

[39] Guo H Li W Nie Z Zhang X Jiao M Bai S . Microinvasive pars plana vitrectomy combined with internal limiting membrane peeling versus anti-VEGF intravitreal injection for treatment-naïve diabetic macular edema (VVV-DME study): study protocol for a randomized controlled trial. Trials. (2023) 24:685. doi: 10.1186/s13063-023-07735-w, 37875997 PMC10594908

[40] Prince J Kumar D Ghosh A Arevalo JF Zhang AY. Surgical Management of Diabetic Macular Edema. Curr Diab Rep. (2023) 23:119–25. doi: 10.1007/s11892-023-01505-3, 37043090

[41] Sun JK Lin MM Lammer J Prager S Sarangi R Silva PS . Disorganization of the retinal inner layers as a predictor of visual acuity in eyes with center-involved diabetic macular edema. JAMA Ophthalmol. (2014) 132:1309–16. doi: 10.1001/jamaophthalmol.2014.2350, 25058813

[42] Das R Spence G Hogg RE Stevenson M Chakravarthy U. Disorganization of inner retina and outer retinal morphology in diabetic macular edema. JAMA Ophthalmol. (2018) 136:202–8. doi: 10.1001/jamaophthalmol.2017.6256, 29327033 PMC5838716

[43] Moein HR Novais EA Rebhun CB Cole ED Louzada RN Witkin AJ . Optical coherence tomography angiography to detect macular capillary ischemia in patients with inner retinal changes after resolved diabetic macular edema. Retina. (2018) 38:2277–84. doi: 10.1097/IAE.0000000000001902, 29068912

[44] Cennamo G Montorio D Fossataro F Fossataro C Tranfa F. Evaluation of vessel density in disorganization of retinal inner layers after resolved diabetic macular edema using optical coherence tomography angiography. PLoS One. (2021) 16:e0244789. doi: 10.1371/journal.pone.0244789, 33434213 PMC7802961

[45] Tripathi A Gaur S Agarwal R Singh N Singh A Parveen S . Disorganization of retinal inner layers as an optical coherence tomography biomarker in diabetic retinopathy: a review. Indian J Ophthalmol. (2025) 73:1245–50. doi: 10.4103/IJO.IJO_800_25, 40880142 PMC12448493

[46] Mathurkar S Daigavane S Saldanha C. A comparative study of ganglion cell complex thickness changes in diabetic macular edema and central retinal vein occlusion macular edema: an optical coherence tomography study. Cureus. (2022) 14:e30609. doi: 10.7759/cureus.30609, 36426324 PMC9680978

[47] Condelipes A Correia D Fernandes I Silva T Correia E Pereira B . Thickness profile of the ganglion cell complex and choroid in patients with persistent diabetic macular edema. Comput Biol Med. (2025) 198:111192. doi: 10.1016/j.compbiomed.2025.111192, 41101191

[48] Maheshwary AS Oster SF Yuson RMS Cheng L Mojana F Freeman WR. The association between percent disruption of the photoreceptor inner segment-outer segment junction and visual acuity in diabetic macular edema. Am J Ophthalmol. (2010) 150:63–67.e1. doi: 10.1016/j.ajo.2010.01.039, 20451897 PMC2900476

[49] Santos AR Costa MÂ Schwartz C Alves D Figueira J Silva R . Optical coherence tomography baseline predictors for initial best-corrected visual acuity response to intravitreal anti-vascular endothelial growth factor treatment in eyes with diabetic macular edema: the Chartres study. Retina. (2018) 38:1110–9. doi: 10.1097/IAE.0000000000001687, 28613220

[50] Kessler LJ Auffarth GU Bagautdinov D Khoramnia R. Ellipsoid zone integrity and visual acuity changes during diabetic macular edema therapy: a longitudinal study. J Diabetes Res. (2021) 2021:1–10. doi: 10.1155/2021/8117650, 34660813 PMC8516551

[51] Saxena S Meyer CH Akduman L. External limiting membrane and ellipsoid zone structural integrity in diabetic macular edema. Eur J Ophthalmol. (2022) 32:15–6. doi: 10.1177/11206721211026106, 34132138

[52] Tsai MJ Cheng CK. Patterns of ellipsoid zone change associated with visual outcome for diabetic macular oedema. Clin Exp Optom. (2022) 105:48–54. doi: 10.1080/08164622.2021.1896333, 33780648

[53] Abd Elhamid AH. Quantitative assessment of outer retinal layer and photoreceptor outer segment layer and their relation to visual acuity in diabetic macular edema. J Ophthalmol. (2019) 2019:1–5. doi: 10.1155/2019/8216150, 30906587 PMC6397985

[54] Ozkaya A Alkin Z Karakucuk Y Karatas G Korhan Fazil M Erdogan G . Thickness of the retinal photoreceptor outer segment layer in healthy volunteers and in patients with diabetes mellitus without retinopathy, diabetic retinopathy, or diabetic macular edema. Saudi J Ophthalmol. (2017) 31:69–75. doi: 10.1016/j.sjopt.2016.12.006, 28559716 PMC5436385

[55] Sardana A Singh K Singh A Singh VK. Optical coherence tomography biomarkers DROL, PROS, SND, hyperreflective walls of foveal cystoid spaces as predictors of central macular thickness and visual acuity in diabetic macular edema treated with intravitreal ranibizumab. Indian J Ophthalmol. (2024) 72:722–7. doi: 10.4103/IJO.IJO_903_23, 38648434 PMC11168529

[56] Upadhyaya A Narula R Takkar B. Structural and functional improvement in persistent macular edema following internal limiting membrane peeling coupled with integrated optical coherence tomography-guided cyst puncture of Intracystic Hyperreflective material. J Vitreoretin Dis. (2025):24741264251383408. doi: 10.1177/24741264251383408, 41180149 PMC12578614

[57] Venkatesh R Sangai S Reddy NG Sridharan A Pereira A Aseem A . Intracystic hyperreflective material in Centre-involving diabetic macular oedema. Graefes Arch Clin Exp Ophthalmol. (2021) 259:2533–44. doi: 10.1007/s00417-021-05083-6, 33710472

[58] Huang XL Song YP Ding Q Chen X Hong L. Evaluation of outer retinal tubulations in diabetic macular edema underwent anti-VEGF treatment. Int J Ophthalmol. (2019) 12:442–50. doi: 10.18240/ijo.2019.03.15, 30918814 PMC6423379

[59] Al-Halafi AM. Outer retinal tubulation in diabetic macular edema following anti-VEGF treatment. Eye Vis (Lond). (2015) 2:9. doi: 10.1186/s40662-015-0018-2, 26613090 PMC4660850

[60] Espina M Arcinue CA Ma F Camacho N Barteselli G Mendoza N . Outer retinal tubulations response to anti-VEGF treatment. Br J Ophthalmol. (2016) 100:819–23. doi: 10.1136/bjophthalmol-2015-307141, 26423451

[61] Mirshahi R Riazi-Esfahani H Khalili Pour E Fadakar K Yarmohamadi P Alemzadeh SA . Differentiating features of OCT angiography in diabetic macular edema. Sci Rep. (2021) 11:23398. doi: 10.1038/s41598-021-02859-y, 34862410 PMC8642537

[62] Basiony AI Mohamed Gad Marey H Ezzat Abdel Fattah AM Aly ZM. Predictive value of optical coherence tomography angiography in management of diabetic macular edema. BMC Ophthalmol. (2024) 24:429. doi: 10.1186/s12886-024-03540-4, 39354390 PMC11445854

[63] Santamaría J Cobos E Biarnes M Caminal JM Rodriguez-Leor R Morwani R . Changes in vessel density patterns assessed with OCTA in patients with diabetic macular edema treated with anti-VEGF therapy. Acta Diabetol. (2024) 61:1385–92. doi: 10.1007/s00592-024-02290-5, 38802603 PMC11531438

[64] Ishibazawa A Nagaoka T Takahashi A Omae T Tani T Sogawa K . Optical coherence tomography angiography in diabetic retinopathy: a prospective pilot study. Am J Ophthalmol. (2015) 160:35–44.e1. doi: 10.1016/j.ajo.2015.04.021, 25896459

[65] Wessel MM Nair N Aaker GD Ehrlich JR D’Amico DJ Kiss S. Peripheral retinal ischaemia, as evaluated by ultra-widefield fluorescein angiography, is associated with diabetic macular oedema. Br J Ophthalmol. (2012) 96:694–8. doi: 10.1136/bjophthalmol-2011-300774, 22423055 PMC3329634

[66] Silva PS Marcus DM Liu D Aiello LP Antoszyk A Elman M . Association of Ultra-Widefield Fluorescein Angiography–Identified Retinal Nonperfusion and the risk of diabetic retinopathy worsening over time. JAMA Ophthalmol. (2022) 140:936–45. doi: 10.1001/jamaophthalmol.2022.3130, 35980610 PMC9389436

[67] Patel RD Messner LV Teitelbaum B Michel KA Hariprasad SM. Characterization of ischemic index using ultra-widefield fluorescein angiography in patients with focal and diffuse recalcitrant diabetic macular edema. Am J Ophthalmol. (2013) 155:1038–1044.e2. doi: 10.1016/j.ajo.2013.01.007, 23453693

[68] Chiku Y Hirano T Hoshiyama K Murata T. Evaluation of retinal edema in nonproliferative diabetic retinopathy using Widefield swept-source OCT. Ophthalmol Sci. (2025) 5:100888. doi: 10.1016/j.xops.2025.100888, 40917263 PMC12410537

[69] Jiang AC Sevgi DD Mugnaini C Whitney J Srivastava SK Talcott KE . Predictive assessment of quantitative ultra-Widefield angiographic features for future need for anti-VEGF therapy in diabetic eye disease. J Pers Med. (2022) 12:608. doi: 10.3390/jpm12040608, 35455724 PMC9032777

[70] Morel JB Fajnkuchen F Amari F Sritharan N Bloch-Queyrat C Giocanti-Aurégan A. Ultra-wide-field fluorescein angiography assessment of non-perfusion in patients with diabetic retinopathy treated with anti-vascular endothelial growth factor therapy. J Clin Med. (2023) 12:1365. doi: 10.3390/jcm12041365, 36835902 PMC9963628

[71] Rabiolo A Parravano M Querques L Cicinelli MV Carnevali A Sacconi R . Ultra-wide-field fluorescein angiography in diabetic retinopathy: a narrative review. Clin Ophthalmol. (2017) 11:803–7. doi: 10.2147/OPTH.S133637, 28490862 PMC5415004

[72] Panozzo G Cicinelli MV Augustin AJ Battaglia Parodi M Cunha-Vaz J Guarnaccia G . An optical coherence tomography-based grading of diabetic maculopathy proposed by an international expert panel: the European School for Advanced Studies in ophthalmology classification. Eur J Ophthalmol. (2020) 30:8–18. doi: 10.1177/1120672119880394, 31718271

[73] Edington M Connolly J Chong NV. Pharmacokinetics of intravitreal anti-VEGF drugs in vitrectomized versus non-vitrectomized eyes. Expert Opin Drug Metab Toxicol. (2017) 13:1217–24. doi: 10.1080/17425255.2017.1404987, 29134820

[74] Iglicki M Busch C Lanzetta P Sarao V Veritti D Rassu N . Vitrectomized vs non-vitrectomized eyes in DEX implant treatment for DMO-is there any difference? The VITDEX study. Eye (Lond). (2023) 37:280–4. doi: 10.1038/s41433-022-01931-9, 35043004 PMC9873723

[75] Yuan Q Liu Y Gou Y Xu H Gao Y Liu Y . Efficacy and safety of the dexamethasone implant in vitrectomized and nonvitrectomized eyes with diabetic macular edema: a systematic review and meta-analysis. Front Pharmacol. (2022) 13:1029584. doi: 10.3389/fphar.2022.1029584, 36532786 PMC9751612

[76] Ng DSC Ruamviboonsuk P Apte RS Bajimaya S Chan CKM Chang A . International consensuses and controversies on causes, diagnosis and management of diabetic macular edema (DME). Prog Retin Eye Res. (2025) 109:101406. doi: 10.1016/j.preteyeres.2025.101406, 41005472

[77] Simó R Hernández C Porta M Bandello F Grauslund J Harding SP . Effects of topically administered neuroprotective drugs in early stages of diabetic retinopathy: results of the EUROCONDOR clinical trial. Diabetes. (2019) 68:457–63. doi: 10.2337/db18-0682, 30389750

[78] Cukras CA Petrou P Chew EY Meyerle CB Wong WT. Oral minocycline for the treatment of diabetic macular edema (DME): results of a phase I/II clinical study. Invest Ophthalmol Vis Sci. (2012) 53:3865–74. doi: 10.1167/iovs.11-9413, 22589436 PMC3390218

[79] PKC-DRS2 Group Aiello LP Davis MD Girach A Kles KA Milton RC . Effect of ruboxistaurin on visual loss in patients with diabetic retinopathy. Ophthalmology. (2006) 113:2221–30. doi: 10.1016/j.ophtha.2006.07.032, 16989901

[80] Miloudi K Oubaha M Ménard C Dejda A Guber V Cagnone G . NOTCH1 signaling induces pathological vascular permeability in diabetic retinopathy. Proc Natl Acad Sci USA. (2019) 116:4538–47. doi: 10.1073/pnas.1814711116, 30787185 PMC6410871

[81] Sheth VS Schlottmann P Lai TYY Abreu F Chang A Eter N . Four-year outcomes of Faricimab in diabetic macular edema: results from the RHONE-X extension trial. Ophthalmology. (2026) 133:599–612. doi: 10.1016/j.ophtha.2026.01.001, 41534798

[82] Zong Y Miyagaki M Yang M Zhang J Zou Y Ohno-Matsui K . Ophthalmic use of targeted biologics in the Management of Intraocular Diseases: current and emerging therapies. Antibodies (Basel). (2024) 13:86. doi: 10.3390/antib13040086, 39449328 PMC11503300

[83] Park SS Bauer G Abedi M Pontow S Panorgias A Jonnal R . Intravitreal autologous bone marrow CD34+ cell therapy for ischemic and degenerative retinal disorders: preliminary phase 1 clinical trial findings. Invest Ophthalmol Vis Sci. (2014) 56:81–9. doi: 10.1167/iovs.14-15415, 25491299 PMC4288143

[84] Eleftheriadou A Riley D Zhao SS Austin P Hernández G Lip GYH . Risk of diabetic retinopathy and diabetic macular oedema with sodium-glucose cotransporter 2 inhibitors and glucagon-like peptide 1 receptor agonists in type 2 diabetes: a real-world data study from a global federated database. Diabetologia. (2024) 67:1271–82. doi: 10.1007/s00125-024-06132-5, 38584180 PMC11153282

